# Job vacancy at the European Centre for Disease Prevention and Control (ECDC)

**DOI:** 10.2807/1560-7917.ES.2026.31.26.260702m

**Published:** 2026-07-02

**Authors:** Eurosurveillance editorial team

**Affiliations:** 1 European Centre for Disease Prevention and Control (ECDC), Stockholm, Sweden

The European Centre for Disease Prevention and Control (ECDC) invites applications for the following positions:

- **Principal Expert ARHAI/Group Leader Scientific Advice and Country Support**
- **Principal Expert/Group Leader Emergency Preparedness and Response**
  The application deadline expires on 18 August 2026.

For more information, please visit the ECDC recruitment platform: https://erecruitment.ecdc.europa.eu/en

